# Protective Efficacy of Selenium in Cisplatin-Induced Retinal Toxicity: An Experimental Immunohistochemical and Ultrastructural Analysis

**DOI:** 10.3390/nu18081236

**Published:** 2026-04-14

**Authors:** Ioannis Konstantinidis, Sophia Tsokkou, Pavlos Pavlidis, Kyriaki Papadopoulou, Dimitrios Kavvadas, Vasilis-Spyridon Tseriotis, Georgios Delis, Chrysanthi Sardeli, Dimitrios Kouvelas, Antonia Siogka, Theodora Papamitsou, Sofia Karachrysafi

**Affiliations:** 1 Laboratory of Histology-Embryology, Department of Medicine, Faculty of Health Sciences, Aristotle University of Thessaloniki, 54124 Thessaloniki, Greece; stsokkou@auth.gr (S.T.); kyriakinp@auth.gr (K.P.); kavvadas@auth.gr (D.K.); sioga@auth.gr (A.S.); 2 Department of Otorhinolarhingology, Head and Neck Surgery, University Medical Center Mainz, 55131 Mainz, Germany; papavlid@googlemail.com; 3 Department of Clinical Pharmacology, School of Medicine, Faculty of Health Sciences, Aristotle University of Thessaloniki, 54124 Thessaloniki, Greece; vasilis-spyridon.tseriotis.24@ucl.ac.uk (V.-S.T.); sardeli@auth.gr (C.S.); kouvelas@auth.gr (D.K.); 4 Department of Neurology, Agios Pavlos General Hospital of Thessaloniki, 55134 Thessaloniki, Greece; 5 Laboratory of Pharmacology, School of Veterinary Medicine, Faculty of Health Sciences, Aristotle University of Thessaloniki, 54124 Thessaloniki, Greece; delis@vet.auth.gr

**Keywords:** cisplatin, selenium, sodium selenite, retinal toxicity, photoreceptor degeneration, oxidative stress, chemotherapy, Transmission Electron Microscopy (TEM), immunohistochemistry, neuroprotection

## Abstract

**Background/Objectives**: Cisplatin is a potent chemotherapeutic agent whose clinical utility is limited by severe side effects, including neurotoxicity affecting the ocular system. The pathophysiology involves oxidative stress and mitochondrial dysfunction, to which the retina is particularly vulnerable. Selenium (Se), an essential trace element and component of antioxidant enzymes, has shown potential in mitigating cisplatin toxicity, although its efficacy with respect to retinal structure and the influence of administration routes remain underexplored. This study aimed to evaluate the protective efficacy of selenium against cisplatin-induced retinal toxicity and compare the effects of intraperitoneal and oral selenium administration. **Methods**: Forty adult male Wistar rats were randomized into four groups (n = 10 each): Group A (Cisplatin Monotherapy, 3.5 mg/kg IP for 5 days; cumulative dose 17.5 mg/kg); Group B (Cisplatin + Intraperitoneal Selenium, 2.73 mg/kg; cumulative dose 60 mg/kg); Group C (Control); and Group D (Cisplatin + Oral Selenium). Selenium prophylaxis, administered as sodium selenite (Na_2_SeO_3_), began two days prior to cisplatin administration and continued for 15 days post-treatment. Retinal evaluation two weeks after cisplatin cessation included light microscopy, semi-quantitative immunohistochemical (IHC) analysis for inflammatory (IL-6) and fibrotic (TGF-β2) markers, and Transmission Electron Microscopy (TEM) for ultrastructural analysis, which were the primary endpoints. Statistical differences in the IHC scores were analyzed via the Kruskal-Wallis H test with Dunn’s post hoc comparisons. **Results**: Cisplatin monotherapy (Group A) caused severe disruption of the retinal architecture, including edema, reactive gliosis, and significant upregulation of IL-6 and TGF-β2. Ultrastructural analysis revealed mitochondrial swelling (cristolysis) and photoreceptor disk fragmentation. Intraperitoneal selenium (Group B) was associated with significant structural preservation and intact mitochondria, with TGF-β2 levels comparable to those of the controls, although the IL-6 level remained moderately elevated. Conversely, oral selenium (Group D) suppressed both IL-6 and TGF-β2 expression to near-negative levels but provided less ultrastructural protection, resulting in persistent mitochondrial swelling and focal photoreceptor disruption. **Conclusions**: Systemic cisplatin induces severe subcellular retinal toxicity characterized by mitochondrial damage and photoreceptor degeneration. Selenium supplementation attenuates these effects; however, outcome patterns differ by administration route. Intraperitoneal selenium was associated with greater morphological and ultrastructural preservation despite persistent IL-6 elevation, whereas oral selenium normalized immunohistochemical marker expression to near-control levels but was associated with more pronounced residual subcellular damage on qualitative TEM assessment. These preliminary morphological and immunohistochemical findings suggest that the route of selenium delivery may influence its neuroprotective profile; however, pharmacokinetic measurements and functional retinal assessments, such as electroretinography, are warranted before its clinical translation.

## 1. Introduction

The advent of platinum-based chemotherapy has fundamentally altered the oncological landscape, transforming the prognosis of previously fatal malignancies into manageable and often curable conditions. Cisplatin (cis-diamminedichloroplatinum(II), MF-Cl_2_H_6_N_2_Pt), a coordination complex of platinum, has revolutionized the treatment of testicular and ovarian cancers and is currently utilized for a broad range of malignancies, including bladder, head and neck, and non-small cell lung cancers [1]. Despite the development of newer generations of platinum derivatives, such as carboplatin and oxaliplatin, cisplatin retains a unique therapeutic profile that necessitates its continued use in first-line protocols [1]. However, the clinical utility of cisplatin is inextricably linked to its formidable toxicity profile, which imposes a dose-limiting ceiling on therapeutic regimens [1,2,3,4,5,6]. While nephrotoxicity and ototoxicity are the most frequently cited adverse events, cisplatin-induced neurotoxicity, which specifically affects the ocular system, represents a significant, often irreversible, source of patient morbidity that severely compromises quality of life [1,2,3,4,5,6,7,8,9].

The pathophysiology of cisplatin-induced cytotoxicity is multifaceted and involves the formation of DNA adducts, mitochondrial dysfunction and the induction of oxidative stress. Upon administration, cisplatin undergoes hydrolysis in the low-chloride intracellular environment, forming highly reactive aquated species [1,2,3,10,11,12,13]. This electrophile preferentially targets the N7 position of guanine residues in nuclear DNA, forming intrastrand and interstrand crosslinks that arrest the cell cycle and trigger apoptosis [1,2,3,10,11,12,13]. While this mechanism is the basis of its antineoplastic activity, the lack of tumor specificity results in collateral damage to rapidly dividing normal tissues and, critically, to postmitotic tissues with high metabolic demands, such as the neural retina. The retina is particularly vulnerable to oxidative insults because of its high consumption of oxygen, abundant concentration of polyunsaturated fatty acids in photoreceptor outer segments, and constant exposure to photooxidative stress. The accumulation of platinum within ocular tissues disrupts the blood-retina barrier and triggers a cascade of inflammatory and apoptotic events that manifest clinically as optic neuritis, papilledema, and progressive visual field loss [1,2,3].

To appreciate the nuances of cisplatin toxicity, a thorough understanding of retinal histology is essential. The retina is a highly organized, laminated extension of the central nervous system, comprising ten distinct layers that facilitate the transduction of light into electrochemical signals [14]. Light traverses the inner layers to reach the photoreceptor layer, where photons are absorbed by the membranous disks of rods and cones [15,16,17,18,19,20,21,22,23]. These photoreceptors synapse with bipolar cells in the outer plexiform layer, which in turn relay signals to retinal ganglion cells (RGCs) in the inner plexiform layer. The axons of RGCs form the nerve fiber layer, which converges to form the optic nerve [16,17,18,19,20,22,23,24,25]. Each of these cellular compartments presents a unique vulnerability profile. The photoreceptor inner segments are densely packed with mitochondria to fuel the Na^+^/K^+^ pumps required for dark current maintenance [15,16,17,18,19,20,21,22,23], making them prime targets for mitochondrial toxins such as cisplatin [15,16,17,18,19,20,21,22,23]. Concurrently, the retinal pigment epithelium (RPE), which is responsible for phagocytosing spent photoreceptor disks and maintaining the blood-retina barrier, accumulates heavy metals, leading to secondary degeneration of the neurosensory retina [16,17,18,19,20,21,22,23,26,27].

The retinal response to cytotoxic injury involves a complex interplay of inflammatory cytokines, especially interleukin-6 (IL-6). IL-6 is a pleiotropic glycoprotein that functions at the interface of immunity and tissue repair. In the context of retinal pathology, IL-6 exhibits a dualistic nature [28,29]. On the one hand, it acts as a pro-inflammatory mediator and is upregulated in response to oxidative stress and cell death. It signals through a hexameric complex involving two molecules each of IL-6, the IL-6 receptor (IL-6R) and the signal transducer gp130 [28,29,30]. The ubiquitous expression of gp130 contrasts with the restricted expression of IL-6R, which is found primarily on hepatocytes and leukocytes [28,29]. However, cells lacking membrane-bound IL-6R, such as endothelial cells, can be activated via “trans-signaling,” where IL-6 binds to a soluble form of its receptor (sIL-6R) [29,31,32]. This pathway is strongly associated with chronic inflammation and pathological neovascularization, phenomena observed in diabetic retinopathy and potentially in chemotherapy-induced vasculopathy [33,34,35,36,37,38]. Conversely, IL-6 possesses potent neurotrophic properties. In the injured central nervous system, IL-6 promotes the survival of neurons and the regeneration of axons [39]. Specifically, signaling through the gp130 receptor has been identified as a critical factor in the regeneration of retinal ganglion cells following optic nerve injury [39]. Thus, the upregulation of IL-6 following cisplatin exposure may represent not only a marker of damage but also a compensatory, albeit insufficient, attempt by retinal tissue to initiate repair and maintain neuronal viability in the face of platinum-induced stress.

Transforming Growth Factor-beta (TGF-β) represents another critical axis in retinal homeostasis and pathology. The TGF-β superfamily governs diverse cellular processes, including proliferation, differentiation and apoptosis [40]. In the eye, the TGF-β2 isoform is predominant and is mainly localized to the vitreous, RPE and choroid [41,42,43,44,45]. TGF-β signaling is mediated by type I and type II serine-threonine kinase receptors, which phosphorylate SMAD proteins to regulate gene transcription [40,46,47,48]. Under physiological conditions, TGF-β maintains tissue quiescence and suppresses inflammation. However, under pathological conditions, such as those induced by chronic oxidative stress, TGF-β drives fibrosis, epithelial-mesenchymal transition (EMT) and reactive gliosis [46,49,50]. In the retina, Müller glial cells respond to injury by upregulating TGF-β, leading to the formation of glial scars that can impede neuronal regeneration and disrupt retinal architecture [43,51,52,53]. The interplay between the pro-survival signals of IL-6 and the pro-fibrotic signals of TGF-β likely defines the ultimate structural outcome of cisplatin toxicity.

Given that the primary mechanism of cisplatin-induced ototoxicity and nephrotoxicity involves the depletion of endogenous antioxidants and the generation of reactive oxygen species (ROS), therapeutic strategies have increasingly focused on antioxidant supplementation. Selenium (Se), an essential trace element, has emerged as a promising candidate [54,55,56,57,58]. Unlike direct radical scavengers, selenium functions as an integral component of the catalytic center of selenoproteins, most notably glutathione peroxidases (GPx) and thioredoxin reductases (TrxR) [54,55,56,57,58]. These enzymes constitute the primary cellular defense against hydrogen peroxide and organic hydroperoxides, preventing the lipid peroxidation that devastates cellular membranes.

The rationale for selenium supplementation in cisplatin-induced toxicity is robust. Cisplatin treatment is known to decrease the activity of antioxidant enzymes and deplete intracellular glutathione. Selenium supplementation can restore these defenses, potentially intercepting the oxidative cascade before irreversible mitochondrial damage occurs. Furthermore, selenium has immunomodulatory effects, enhancing the activity of natural killer cells [54,59,60] and regulating the production of cytokines, which may help modulate the inflammatory response described above [54,61].

However, the pharmacokinetics of selenium are complex. Orally absorbed selenium is transported to the liver, where it is incorporated into Selenoprotein P (SELENOP, formerly Sepp1), a multi-functional glycoprotein that serves as the principal selenium transport protein in plasma. SELENOP delivers selenium to peripheral tissues, including the brain and other neural structures, via apolipoprotein E receptor-2 (ApoER2)-mediated endocytosis [54,62,63,64,65,66]. Hepatocyte-derived SELENOP is central to whole-body selenium homeostasis: it retains selenium within the organism and distributes it from the liver to extra-hepatic tissues, with prioritization governed by tissue-specific receptor expression [54,62,63,64,65,66]. This SELENOP-dependent transport system raises critical questions about the bioavailability of oral selenium preparations in acute therapeutic windows. While oral administration ensures steady-state selenium distribution via the SELENOP cycle, intraperitoneal delivery may bypass this rate-limiting hepatic processing step, potentially achieving higher acute tissue concentrations. This mechanistic distinction has direct relevance to acute cytoprotective strategies during chemotherapy cycles.

The clinical relevance of selenium status in tissues vulnerable to oxidative injury has been further underscored by a recent clinical trial, which demonstrated that serum selenium, zinc, and total antioxidant status (TAS) are significantly altered in colorectal neoplasia, highlighting the broader importance of trace element adequacy in cancer patients [67]. Moreover, prospective data from the European Prospective Investigation into Cancer and Nutrition (EPIC) cohort revealed that higher serum selenium and SELENOP levels are inversely associated with colorectal cancer risk, providing population-level evidence that selenium transport and retention are biologically significant determinants of disease susceptibility [68].

While the nephroprotective and hepatoprotective effects of selenium are well-documented, its efficacy in preventing cisplatin-induced retinal toxicity remains underexplored [69]. The selection of IL-6 and TGF-β2 as immunohistochemical markers in this study was based on their complementary roles in the retinal response to injury. IL-6 represents the acute inflammatory axis and has a well-characterized dual nature, acting as both a pro-inflammatory mediator and a neurotrophic signal via gp130-dependent pathways, making it particularly informative in neuroretinal injury models. TGF-β2, as the predominant ocular isoform of the TGF-β superfamily, captures the fibrotic and gliotic remodeling response that determines long-term structural outcomes. Together, these markers provide an integrated view of the inflammatory-fibrotic cascade. Critically, no previous study has compared intraperitoneal and oral selenium administration in a model of cisplatin-induced retinal toxicity via an integrated approach combining immunohistochemical markers and transmission Electron Microscopy. The novelty of the present investigation therefore lies not in demonstrating a protective role for selenium per se but in the systematic, within-experiment comparison of two clinically distinct routes of administration, assessed through complementary endpoints, semi-quantitative IHC scoring for IL-6 and TGF-β2, and qualitative ultrastructural evaluation of photoreceptor and mitochondrial integrity via TEM, within the same experimental framework. This approach reveals a previously unreported dissociation between cytokine-level suppression and subcellular structural preservation that has direct implications for the design of future cytoprotective trials in oncology.

## 2. Materials and Methods

### 2.1. Experimental Unit

The study utilized forty adult male Wistar rats, aged between 9 and 12 weeks and weighing between 300 and 400 g. The Wistar strain was selected because of its established use in toxicological models and the anatomical similarity of its ocular structures to those of the human eye, facilitating translational inference [70,71,72,73]. The animals were sourced from a certified breeding facility (EL-54-BIOexp-04) to ensure genetic homogeneity.

### 2.2. Housing and Care of the Animals

Upon arrival, the animals were acclimatized for a period of seven days. They were housed in custom-fabricated plexiglass cages designed to minimize environmental stress. The facility environment was strictly controlled, with the ambient temperature maintained at 21–22 °C and the relative humidity maintained above 50%. A standard 12 h light, 12 h dark photoperiod was enforced via automated timers. This circadian regulation is critical in ophthalmological studies, as retinal physiology, including photoreceptor disk shedding and renewal, is tightly coupled to the light cycle. The animals were housed in a sound-attenuated room to prevent stress-induced physiological alterations. Access to standard rodent chow and water was provided ad libitum throughout the study.

### 2.3. Sample Size and Randomization

In terms of sample size, insufficient data are available in the literature concerning the expression of proinflammatory cytokines (immunohistochemical markers) in the retina, which constitutes the main focus of this study following cisplatin administration. Moreover, data concerning the normal reference ranges for these specific cytokines are lacking. Existing studies using animal models in the contemporary literature that investigate the effects of cisplatin on the retina and compare the potential protective effects of other agents typically employ four groups of ten animals each [70,71,72,73,74,75,76,77].

Randomization was performed by a designated researcher (S.K.) via the RAND() function in Microsoft Excel to generate a random number sequence. Each animal was assigned a unique identification number, and the random number sequence was used to determine group allocation. The allocation list was kept in a sealed envelope by S.K. and disclosed only to the primary investigator responsible for drug administration (P.P.), who prepared and administered all the injections. All other personnel, including outcome assessors performing immunohistochemical scoring (A.S. and T.P.) and Electron Microscopy operators, were blinded to group assignments throughout the study. The randomization list was revealed to the study team only after all scoring and data collection were completed.

### 2.4. Study Design

**Figure 1:**
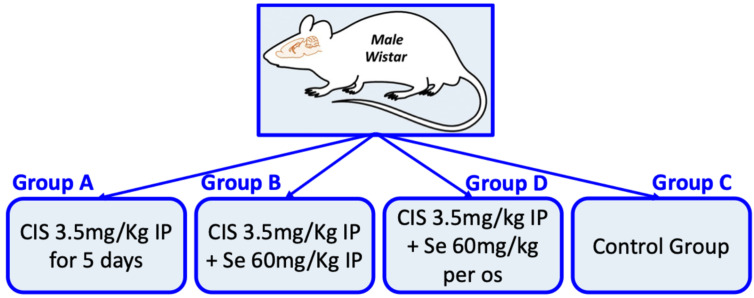
Schematic representation of the experimental protocol. Cisplatin was administered to the groups over a five-day regimen, while selenium administration began two days prior and continued for fifteen days following cisplatin treatment. Notably, selenium was administered at a cumulative dose of 60 mg/kg. Note: CIS: cisplatin, Se: selenium, IP: intraperitoneal administration, per os: oral administration.

The experimental groups were defined as follows (Figure 1):

**Group A (Cisplatin Monotherapy):** This group served as the positive control for toxicity. The animals received cisplatin intraperitoneally at a dose of 3.5 mg/kg body weight daily for five consecutive days. The cumulative dose of 17.5 mg/kg was selected on the basis of established cisplatin neurotoxicity protocols in rodents. Multiday regimens of 2.3–3.5 mg/kg/day for 4–5 days, yielding cumulative doses of 16–23 mg/kg, are widely used to model cisplatin-induced neurotoxicity, including ototoxicity and peripheral neuropathy. This fractionated approach more closely mimics clinical chemotherapy regimens, such as 15–20 mg/m^2^ for 5 days, where repeated low-dose administration produces cumulative organ toxicity, than single high-dose bolus models (7–16 mg/kg), which primarily induce acute nephrotoxicity [74,78,79,80]. The chosen regimen was designed to produce retinal toxicity while maintaining animal viability throughout the observation period.

**Group B (Cisplatin + Intraperitoneal Selenium):** This group investigated the efficacy of parenteral selenium. The animals received the same cisplatin regimen (3.5 mg/kg IP for 5 days) concomitant with the intraperitoneal administration of selenium at a cumulative dose of 60 mg/kg. Importantly, selenium prophylaxis commenced two days prior to the first cisplatin injection and continued for 15 days following the final cisplatin dose. This extended duration aimed to cover the periods of both acute oxidative insult and delayed tissue remodeling.

**Group D (Cisplatin + Oral Selenium):** This group investigated the efficacy of enteral selenium. The animals received cisplatin (3.5 mg/kg IP for 5 days) combined with selenium administered per os (via oral gavage) at a cumulative dose of 60 mg/kg. The timing and duration of selenium administration mirrored those of Group B, allowing for a direct comparison of administration routes while controlling for dosage and timing.

**Group C (Control):** This group served as the negative control to establish baseline retinal morphology and marker expression. The animals received no pharmacological agents and no injections or gavage procedures. All the animals were handled with identical frequency and under the same environmental conditions, as the treatment groups were to control for stress associated with manipulation.

Cisplatin (CISPLATIN/HOSPIRA, Pfizer Hellas S.A., Athens, Greece) was obtained as a sterile solution at a concentration of 1 mg/mL suitable for injection. Sodium selenite (Na_2_SeO_3_, 99%; Sigma-Aldrich, St. Louis, MO, USA; product no. 214485) was used as the selenium source. The compound was freshly dissolved in sterile physiological saline (0.9% NaCl) immediately prior to administration. The solution was vortexed until complete dissolution and protected from light until use. For intraperitoneal (IP) administration, sodium selenite was diluted in sterile saline to achieve the desired concentration and injected at a volume of 1–2 mL/kg body weight. For oral administration (PO), the compound was similarly dissolved in sterile saline and administered via oral gavage, with the volume adjusted to 2–5 mL/kg body weight. Fresh solutions were prepared daily immediately before administration. The rats received sodium selenite once daily for 22 consecutive days either intraperitoneally or orally. The cumulative dose was 60 mg/kg body weight, corresponding to 2.73 mg/kg/day sodium selenite (approximately 1.26 mg elemental selenium/kg/day). This regimen was selected as a fractionated antioxidant-support protocol on the basis of previous experimental evidence showing selenium-mediated protection against cisplatin-induced retinal and neural injury [69].

The co-primary endpoints of this study were: (i) semi-quantitative immunohistochemical staining intensity scores for IL-6 and TGF-β2, which were assessed on an ordinal scale (0–3), and (ii) qualitative ultrastructural assessment of photoreceptor and mitochondrial integrity via TEM. These endpoints were assessed in retinal tissue collected two weeks after the final cisplatin administration, allowing for the evaluation of both acute damage and early remodeling.

### 2.5. Anesthesia, Surgical Procedures and Tissue Fixation

Two weeks after the cessation of the cisplatin protocol, the animals were prepared for tissue collection. Anesthesia was induced via a multimodal protocol to ensure profound analgesia and immobility. Pre-medication involved intramuscular administration of dexmedetomidine (50 μg/kg, CEPEDEX 0.1 mg/mL, CP-Pharma Handelsgesellschaft mbH, Burgdorf, Germany) and butorphanol (0.1 mg/kg, BUTOMIDOR 10 mg/mL, VetViva Richter GmbH, Wels, Austria). Induction was achieved with intramuscular ketamine (25 mg/kg, ANAESTAMINE 100 mg/mL, Le Vet Beheer B.V., Oudewater, The Netherlands) or, where necessary for deeper anesthesia, intravenous propofol (0.5 mg/kg, PROPODINE 10 mg/mL, Dechra Regulatory B.V., Bladel, The Netherlands). This combination ensures stable hemodynamics while providing deep somatic analgesia. Topical anesthesia of the ocular surface was provided by 0.5% proparacaine hydrochloride drops (Alcaine, Alcon, Inc., Geneva, Switzerland), and pupillary dilation was achieved with 1% tropicamide (Bausch & Lomb Ireland Ltd., Waterfold, Ireland) to facilitate gross examination prior to enucleation.

Following the confirmation of deep anesthesia, the animals were euthanized via cervical dislocation. Immediate bilateral enucleation of the ocular globes, including a segment of the optic nerve, was performed in a dedicated ophthalmic surgical suite under aseptic conditions. Rapid collection of tissue is critical for preventing postmortem autolysis, which can mimic toxicological damage, particularly in sensitive photoreceptor layers.

### 2.6. Preparation for Observation Under the Optical Microscope

The retinal samples were cut into blocks 0.5 cm to 4 cm thick, placed in special capsules, and immediately immersed in a 10% formaldehyde fixative solution of 35% formaldehyde. The samples were dehydrated and clarified for paraffin embedding. Dehydration was performed in an ascending alcohol series for six hours (76%, 96%, 100%, and 100%), followed by a 4 h clarification in xylol. The capsules were then soaked in liquid paraffin for four hours [18,20,81,82,83]. The tissues were placed in special metal molds with liquid paraffin and cooled at 4 °C for 20 min. Each paraffin cube was sectioned with a semiautomatic microtome (Leica RM2255, Leica, Wetzlar, Germany) at 3 μm, resulting in 10 incisions. From these sections, the first three were mounted on slides, while the remaining seven were placed on positively charged slides and dried at room temperature for one hour. The first 3 incisions were placed in a furnace for one hour at 65 degrees Celsius, deparaffinized in xylol for 10 min, and then hydrated in descending ethanol for twenty minutes (100%, 100%, 96%, and 76%). They were stained with hematoxylin for five minutes, rinsed in tap water for another five minutes, briefly differentiated with 1% solution for one second, stained with eosin for one minute, dehydrated for five minutes in ethanol, and placed in xylol for clarification for another five minutes. Finally, the slides were covered with “balm of Canada” for microscopy. The remaining positively charged slides were subjected to immunohistochemistry with IL-6 (Santa Cruz, CA, USA; 1:100 dilution) and TGF-β2 (Abcam, Cambridge, UK; 1:100 dilution). The sections were incubated in a humidified chamber with 200 μL of IL-6 antibody at a dilution of 1:100 for 1 h or with TGF-β2 at a dilution of 1:100 for 30 min. The samples were subsequently rinsed for 5 min with wash buffer, incubated with peroxidase blocking solution for 10 min, rinsed again for 5 min, and incubated with Dako Polymer EnVision HRP (Agilent Technologies, Santa Clara, CA, USA) for 25 min at room temperature. The samples were subsequently rinsed with wash buffer for 5 min, and 3–4 drops of freshly prepared DAB chromogen (Sigma–Aldrich, St. Louis, MO, USA) were added to the samples, which were then incubated for 10 min at room temperature. Moreover, they were rinsed with distilled water and wash buffer for 5 min, respectively. Additionally, 3–4 drops of hematoxylin were added to the sample, which was subsequently incubated for 2 min at room temperature. Again, wash buffer and distilled water were used for rinsing for 5 min. Finally, dehydration was performed in an ascending series of alcohols, immersion in xylol, and coverage with a coating agent. The sample was observed under an optical microscope for diagnosis. Therefore, eosin–hematoxylin staining and immunohistochemical staining for the immunohistochemical markers IL-6 and TGF-β2 were performed for each subgroup.

### 2.7. Double Blind Assessment

All the slides were examined under an optical microscope by two independent professional observers (A.S. and T.P.), both of whom have extensive experience in the immunohistochemical evaluation of retinal tissue. The observers were blinded to the rat identification number and group assignments. To eliminate bias, assessments were conducted independently and recorded electronically before consensus discussion. Immunohistochemical staining intensity was assessed as the primary outcome via a semiquantitative ordinal scale: negative (0, −), mild (1, +), moderate (2, ++), and intense (3, +++). For each animal, three sections were evaluated per marker (IL-6 or TGF-β2). Within each section, five nonoverlapping high-power fields (×40) were assessed and selected systematically from the central retina to ensure representative sampling. The final score for each animal represents the consensus modal score across all evaluated fields.

### 2.8. Preparation for Observation Under the Electron Microscope

Retinal tissue samples were dissected into pieces smaller than <1 cm^3^, fixed in 3% glutaraldehyde for 2 h, rinsed in phosphate buffer for 10′, and postfixed in 1% osmium tetroxide (OsO4) for 1/2 h. Samples were washed with phosphate buffer for 10′ and distilled twice for 10′. The samples were simultaneously fixed with 1% uranyl acetate for 16 h and then dehydrated with an ascending series of alcohols (30° for 5′, 50° for 5′, 70° for 5′, 96° for 5′, and 100° for 5′ × 6 times) [18,20]. The samples were encapsulated in Epon resin, and very thin incisions (60–90 nm) were made. Finally, the sections were subjected to Reynold’s stain and observed via transmission Electron Microscopy (TEM) with a JEOL 1011 electron microscope at 80 kV (JEOL-Tokyo, Tokyo, Japan).

For ultrastructural analysis, a minimum of 10 non-overlapping electron micrographs per animal were captured at magnifications of ×12,000 and ×24,000, systematically sampling from the photoreceptor layer (inner and outer segments) and adjacent retinal layers. Images were evaluated qualitatively for key ultrastructural parameters: photoreceptor outer segment disk integrity (intact, focal disruption, or extensive fragmentation), mitochondrial morphology in the inner segment (normal cristae, mild swelling, or cristolysis), and connecting cilium integrity. Results are reported descriptively based on representative images from each group, and formal quantitative scoring of the TEM findings was not performed.

### 2.9. Ethical Compliance and Animal Welfare

This study was designed and executed in strict adherence to the ARRIVE 2.0 (Animal Research: Reporting of In Vivo Experiments) guidelines to ensure the reproducibility and ethical integrity of the research (Supplementary Table S1) [84]. The experimental protocol received approval from the Veterinary Directorate of PKM, Department of Animal Health & Veterinary Perception, Medicines and Applications (YZ-KAFE), under protocol number 663613 (3000), dated 20 September 2022. All animal care, handling, and surgical procedures complied with the European Union Directive 2010/63/EU on the protection of animals used for scientific purposes.

Animals were monitored twice daily during the cisplatin administration phase (Days 1–5) and once daily thereafter for general health status. The monitoring parameters included body weight (recorded every 48 h), food and water intake, coat condition, posture, mobility, respiratory pattern, and the presence of diarrhea, piloerection, or signs of distress. Predefined humane endpoints included ≥20% loss of baseline body weight, persistent hunched posture with immobility for >6 h, labored breathing, self-mutilation, or inability to reach food/water. Animals meeting the humane endpoint criteria were euthanized immediately. No animals met the humane endpoint criteria during the study. No unexpected adverse events were recorded. All 40 animals completed the study protocol. Both eyes from each animal were collected; retinal tissue from all 10 animals per group was included in the IHC analyses, and TEM was performed on representative samples from each group. No animals or data points were excluded from the analyses.

### 2.10. Statistical Analysis

Statistical analysis was conducted using the raw ordinal scoring data from the immunohistochemical evaluation. Owing to the nonparametric nature of the scoring scale (0–3) and the nonnormal distribution of the data, nonparametric statistical tests were employed. The Kruskal-Wallis H test was used to determine if statistically significant differences existed among the four independent groups. To calculate effect sizes for each primary outcome, the Eta Squared ($\eta_{H}^{2}$) test for the Kruskal-Wallis test was used. Post hoc pairwise comparisons were performed via Dunn’s test with Bonferroni correction to adjust for multiple comparisons and control for the familywise error rate. A *p* value of less than 0.05 was considered statistically significant. All analyses were performed via IBM SPSS Statistics software version 29.0.

## 3. Results

No mortality or observable clinical signs of toxicity were recorded in any of the experimental groups during the study period. Body weight progression was comparable among the groups. Therefore, all the animals (n = 40) were included in the analyses (Supplementary Figure S1).

### 3.1. Control Group

**Figure 2:**
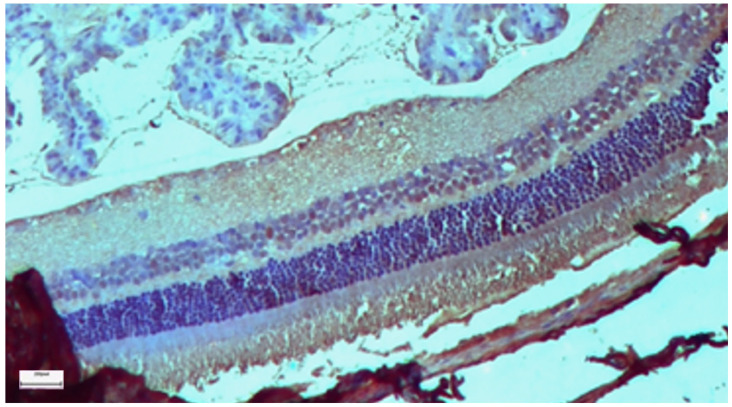
Control Group: Retina without morphological lesions and with negative staining (−). Immunohistochemical analysis (IL-6). ×40. Scale bar: 100 μm.

**Figure 3:**
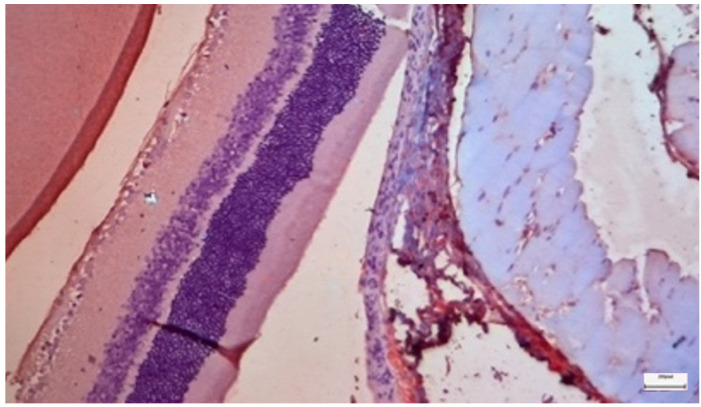
Control Group: Retina without morphological lesions and with negative staining (−). Immunohistochemical analysis (TGF-β2). ×40. Scale bar: 100 μm.

The control group demonstrated the baseline histological features of a healthy mammalian retina. Under light microscopy, the ten layers of the retina were clearly distinguishable and well organized. The retinal pigment epithelium (RPE) appears as a continuous monolayer of cuboidal cells. The photoreceptor outer nuclear layer (ONL) and the inner nuclear layer (INL) showed compact, regular cellular arrangements. Immunohistochemical analysis for both IL-6 (Figure 2) and TGF-β2 (Figure 3) revealed predominantly negative results (score 0), with occasional mild positivity (score 1) observed in 1 of 10 animals for each marker (median 0; range 0–1), representing baseline variability and indicating a lack of constitutive inflammatory or profibrotic signaling in the healthy retina.

**Figure 4:**
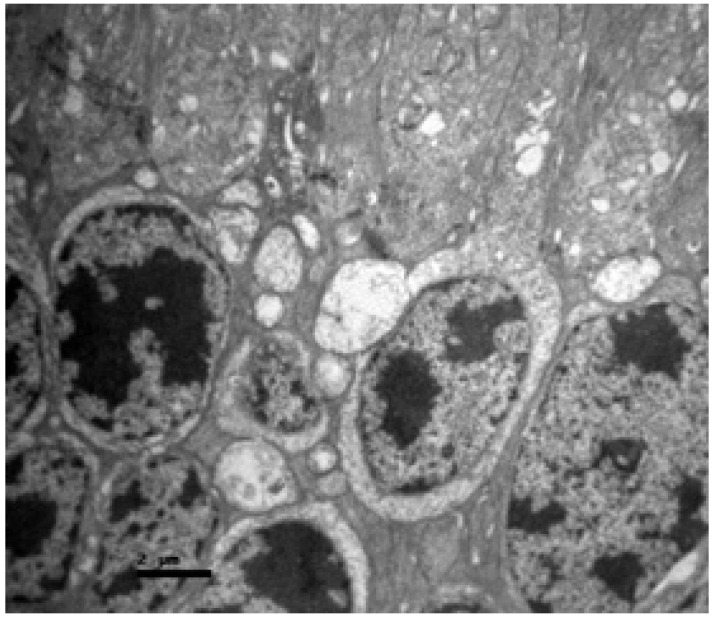
Control Group: Retina without morphological lesions. In the lower half of the image, the cell bodies with their nuclei and nucleoli can be observed, along with some of the axons of the rods and cones arranged in a palisade-like pattern, forming the outer granular–nuclear layer. Transmission Electron Microscopy. ×12,000. Scale bar: 2 μm.

**Figure 5:**
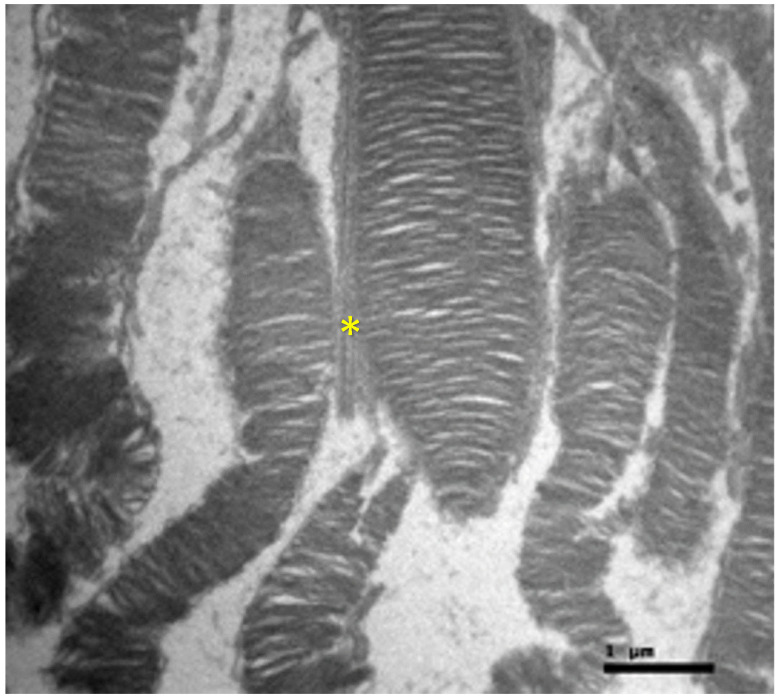
Control Group: Retina without morphological lesions. The outer segments of the rods and cones are observed, displaying a normal architecture of the membranous disks, which consists of multiple parallel stacks of flattened, double-walled, transverse lamellae of the cell membrane, enclosed by an extension of this membrane with a diameter of approximately 1 μm. In the center of the image, a portion of the modified cilium of the connecting stalk (yellow asterisk) of the photoreceptor is visible, showing the characteristic ‘9 + 0’ arrangement, containing nine peripheral pairs of microtubules without the central pair typically encountered. The outer segments of the photoreceptors are in close contact with the microvilli of the apical surface of the cuboidal cells of the retinal pigment epithelium and are embedded within an extracellular matrix rich in proteins, glycoproteins, and glycosaminoglycans. Transmission Electron Microscopy. ×24,000. Scale bar: 1 μm.

Ultrastructural analysis via TEM confirmed these findings (Figure 4 and Figure 5). The photoreceptor outer segments appeared as densely packed, parallel stacks of membranous disks, which are essential for efficient photon capture. The inner segments were rich in mitochondria with well-defined, dense cristae, reflecting the high metabolic capacity required for phototransduction. The connecting cilium, featuring the characteristic 9 + 0 microtubule arrangement, was intact.

### 3.2. Group A (Cisplatin Monotherapy)

The administration of cisplatin resulted in severe disruption of the retinal architecture. Light microscopy revealed a generalized increase in retinal thickness, indicative of intracellular and extracellular edema, particularly within the nuclear layers. The GCL exhibited significant degeneration, characterized by a reduction in neuronal density and the presence of reactive gliosis, a proliferation of glial elements in response to neuronal injury.

**Figure 6:**
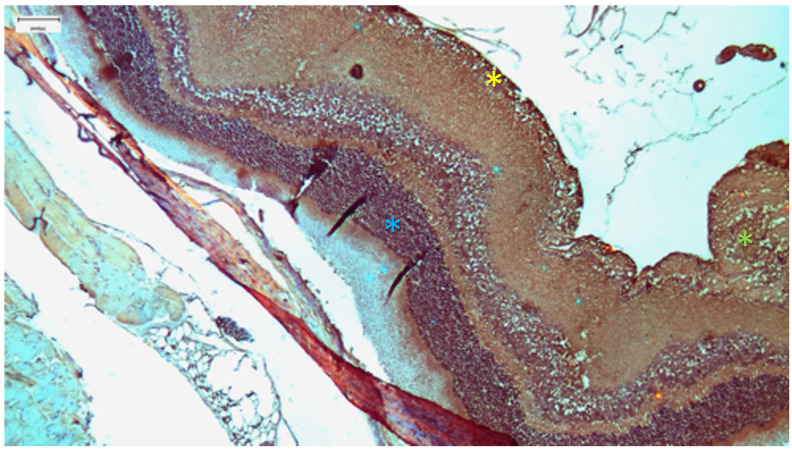
Group A: Retina with increased thickness and disruption of its architecture, particularly within the ganglion cell layer, indicative of gliosis (green asterisk). Moderately positive (++) staining is observed in the inner limiting membrane, the nerve fiber layer and the ganglion cells (yellow asterisk), whereas mildly positive (+) staining is present in the outer nuclear layer and the outer limiting membrane (blue asterisk). Immunohistochemical analysis (IL-6). ×40. Scale bar: 100 μm.

**Figure 7:**
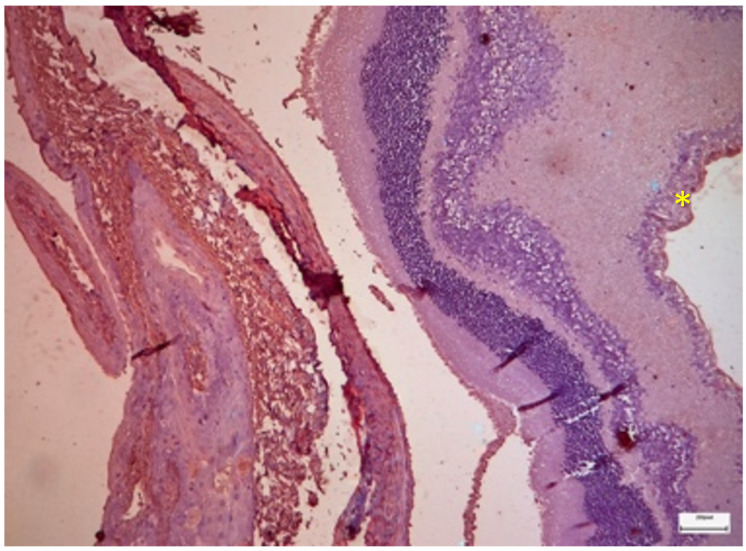
Group A: Retina with increased thickness and architectural disruption, showing mild positive (+) staining in the ganglion cells (yellow asterisk). Immunohistochemical analysis (TGF-β2). ×40. Scale bar: 100 μm.

Immunohistochemical staining revealed marked upregulation of IL-6 (Figure 6). The stain was moderately positive (++) and diffusely localized to the Inner Limiting Membrane (ILM), Nerve Fiber Layer (NFL) and cytoplasm of surviving ganglion cells (median 2.00; IQR 2.00–2.25). Notably, positive staining was also observed in the endothelial cells of the choroidal vasculature (++), suggesting a component of vascular inflammation. TGF-β2 expression was also upregulated (+), primarily within the GCL, which was correlated with the morphological observation of gliosis (median 1.00; IQR 1.00–2.00; Figure 7).

**Figure 8:**
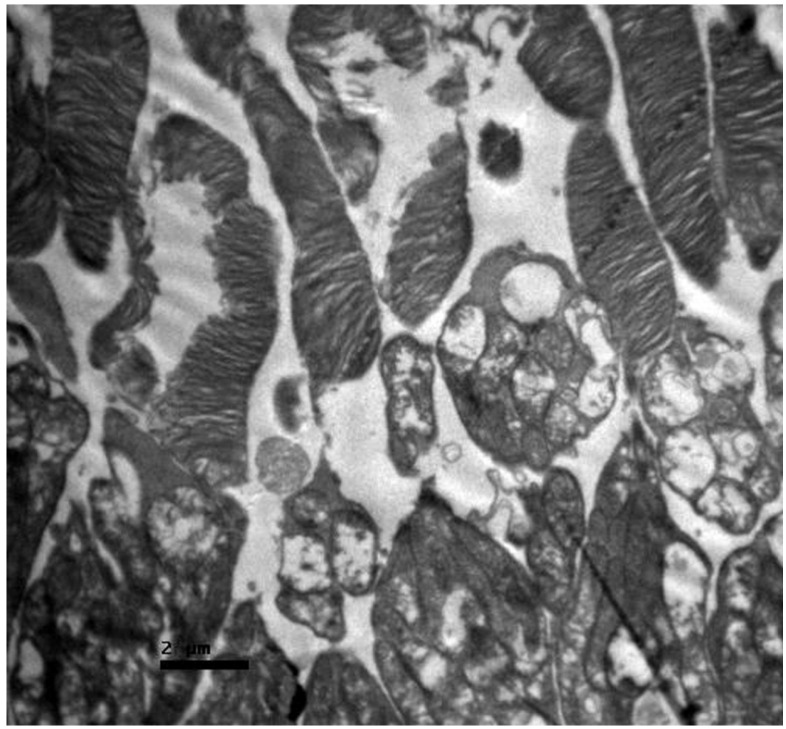
Group A: Retina with severe disruption of the photoreceptors, showing extensive fragmentation of the membranous disks and marked swelling with architectural distortion of the mitochondria in the inner segment. Transmission Electron Microscopy. ×12,000. Scale bar: 2 μm.

Ultrastructurally, the damage was profound (Figure 8). The photoreceptor outer segments exhibited extensive fragmentation and vesiculation of the membranous disks, indicating a complete loss of structural integrity necessary for vision. More critically, the mitochondria within the inner segments appeared swollen and vacuolated, with a loss of crista structure (cristolysis). This mitochondrial swelling is suggestive of permeability transition pore opening and catastrophic energetic failure, which is consistent with the mitochondrial toxicity profile of cisplatin.

### 3.3. Group B (Cisplatin + Intraperitoneal Selenium)

Compared with the cisplatin-only group, the group receiving intraperitoneal selenium prophylaxis presented significant preservation of retinal structure. The retinal thickness was comparable to that of the controls, suggesting an absence of edema. The integrity of the ILM and fibrous layers was maintained.

**Figure 9:**
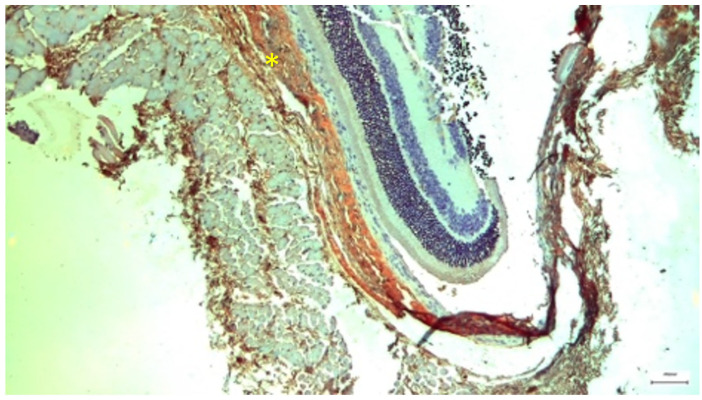
Group B: Retina with normal thickness and no evident architectural disruption, showing mild positive (+) staining in the inner limiting membrane, mild to moderate positive (+/++) staining in the nerve fiber layer and ganglion cell layer, and moderate positive (++) staining in the fibrous and choroidal layers (yellow asterisk). Immunohistochemical analysis (IL-6). ×10. Scale bar: 200 μm.

**Figure 10:**
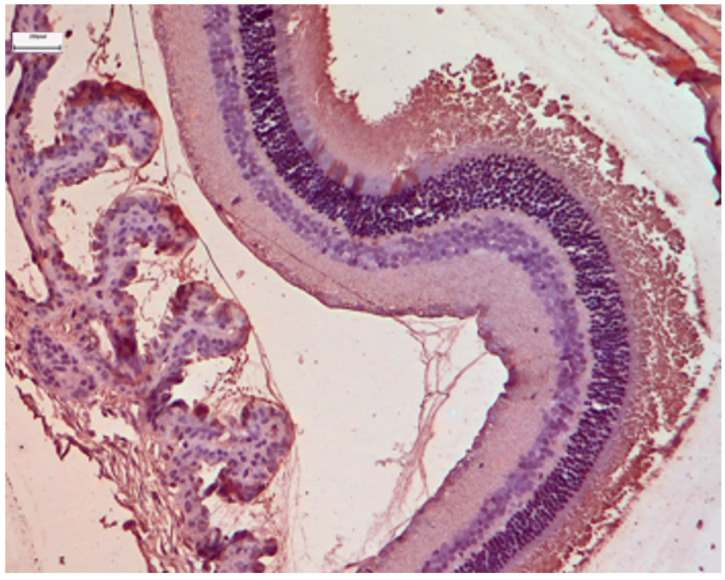
Group B: Retina with normal thickness and negative (−) staining. Immunohistochemical analysis (TGF-β2). ×40. Scale bar: 100 μm.

The immunohistochemical results revealed a nuanced profile (Figure 9). IL-6 expression remained detectable, with mild to moderate staining (+/++) in the GCL and NFL (median 1.50; IQR 1.00–2.00). While significantly elevated compared with that in the controls, the intensity was qualitatively different from the profound upregulation observed in Group A. Interestingly, TGF-β2 expression was largely suppressed (−), suggesting that IP selenium effectively blocked the signaling pathways leading to fibrosis and scarring (Figure 10).

**Figure 11:**
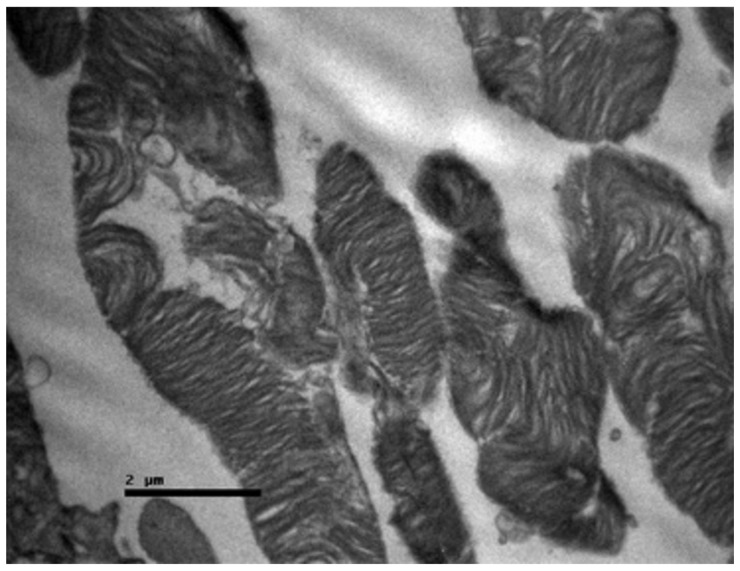
Group B: Retina with mild disruption of the architecture of the photoreceptor outer segments and focal fragmentation of the membranous disks. Transmission Electron Microscopy. ×12,000.

Ultrastructurally, the protective effect was evident (Figure 11). The photoreceptor outer segments showed only mild focal disorganization, with the majority of disk stacks remaining intact. Mitochondrial morphology was largely preserved, with minimal swelling observed compared to Group A, indicating that IP selenium successfully intercepted the mitochondrial oxidative stress cascade.

### 3.4. Group D (Cisplatin + Oral Selenium)

**Figure 12:**
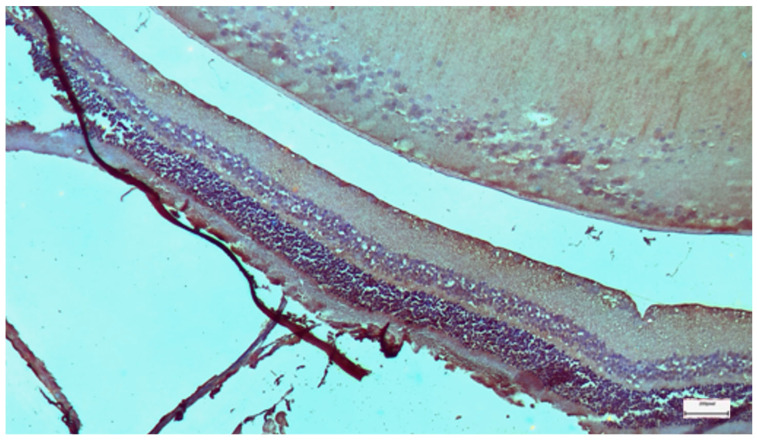
Group D: Retina with normal thickness, without any evident architectural disruption, and with mild positive (+) staining in the ILM. Immunohistochemical analysis (IL-6). ×10. Scale bar: 200 μm.

**Figure 13:**
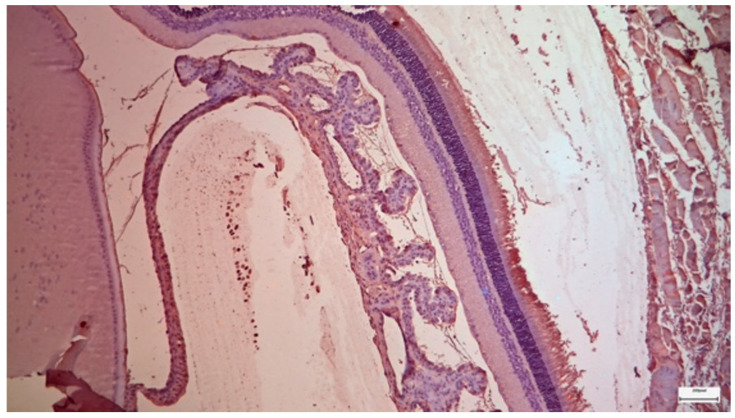
Group D: Retina with normal thickness and negative (−) staining. Immunohistochemical analysis (TGF-β2). ×10. Scale bar: 200 μm.

**Figure 14:**
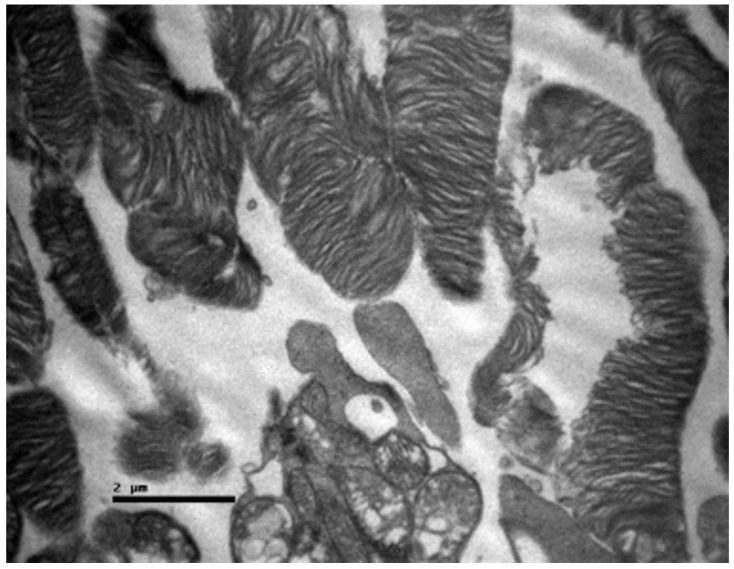
Group D: Retina with mild disruption of the architecture of the photoreceptor outer segments, with focal fragmentation of the membranous disks and moderate swelling of the mitochondria in the outer portion of their inner processes. Transmission Electron Microscopy. ×12,000. Scale bar: 2 μm.

The oral selenium group displayed a distinct phenotype. Compared with those in Group A, the retinal layers in Group A presented more signs of stress than those in Group B did, particularly in the ILM and connective tissues. This group presented the lowest levels of cytokine expression among the treatment groups. IL-6 staining was predominantly negative (0), comparable to that in the control group, with mild positive (1) staining observed in the ILM in a subset of the samples (Figure 12). Similarly, TGF-β2 expression was negligible (−) (Figure 13). However, the ultrastructural findings revealed a dissociation between marker expression and structural integrity (Figure 14). TEM images revealed mild disruption of the architecture of the outer segments of the photoreceptors, with focal fragmentation of the membranous disks and moderate swelling of the mitochondria in the outer segment of their inner processes. Compared with that in the cisplatin-only group, the damage was more pronounced than that in the IP-only group. This pattern reveals a dissociation between cytokine suppression and ultrastructural preservation in the oral selenium group: despite the near-control IHC scores, the TEM findings revealed persistent subcellular damage exceeding that observed in the IP selenium group.

### 3.5. Statistical Analysis of Immunohistochemical Scores

#### 3.5.1. Statistical Analysis of Immunohistochemical Scores for IL-6

Descriptive statistics for the IL-6 immunohistochemical scores (0–3) in each group (n = 10 per group) are shown in Table 1. Overall, Groups A and B presented greater central tendencies and tighter clustering around their medians than did the Control and Group D groups, which both clustered at the low end of the scale.

A Kruskal-Wallis H test was conducted to determine whether there were differences in the IL-6 scores among the four groups. The test revealed a statistically significant difference in median IL-6 scores, χ^2^(3) = 31.37, *p* < 0.001, with a relatively large effect size ($\eta_{H}^{2}=0.788\;(95\%\;CI\;0.621-0.894)$.

In post hoc pairwise comparisons via Dunn’s test with Bonferroni adjustment and calculated effect size ($r\;=\;\frac{\left|Z\right|}{\sqrt{n_{i}+n_{j}}}$) for each significant comparison, both Group A and Group B presented significantly higher IL-6 scores than did the Control group (Control vs. A: adjusted *p* < 0.000, r = 1.09; Control vs. B: adjusted *p* = 0.004, r = 0.77), whereas Group D did not differ from the Control group (adjusted *p* = 1.000). Among the treatment groups, Group A and Group B each scored significantly higher than Group D did (A vs. D: adjusted *p* < 0.000, r = 0.93; B vs. D: adjusted *p* = 0.045, r = 0.60), but there was no significant difference between Group A and Group B (adjusted *p* = 0.860).

#### 3.5.2. Statistical Analysis of Immunohistochemical Scores for TGF-β2

Descriptive statistics for the TGF-β2 immunohistochemical scores (0–3) of each group (n = 10 per group) are presented in Table 2. Overall, Group A displayed the highest central tendency and the tightest clustering around moderate staining levels, whereas Control and Group D remained concentrated at the baseline end of the scale, with Group B occupying an intermediate position.

A Kruskal-Wallis H test was conducted to determine differences in the TGF-β2 scores among the four groups. The test revealed a statistically significant difference in median TGF-β2 scores, χ^2^(3) = 20.10, *p* < 0.001, with a relatively large effect size ($\eta_{H}^{2}=0.475\;(95\%\;CI\;0.245-0.682)$.

In post hoc pairwise comparisons via Dunn’s test with Bonferroni adjustment and calculated effect size ($r\;=\;\frac{\left|Z\right|}{\sqrt{n_{i}+n_{j}}}$) for each significant comparison, Group A presented significantly higher IHC scores than did the Control group (Control vs. A: adjusted *p* < 0.000, r = 0.94), whereas Groups B and D did not differ from the Controls (Control vs. B: adjusted *p* = 1.000; Control vs. D: adjusted *p* = 1.000). Compared with Group B and Group D, Group A scored significantly higher (A vs. D: adjusted *p* = 0.004, r = 0.76; A vs. B: adjusted *p* = 0.017, r = 0.67), but there was no significant difference between Group D and Group B (adjusted *p* = 1.000).

## 4. Discussion

### 4.1. Cisplatin-Induced Retinal Toxicity

The findings of this study provide preliminary morphological and immunohistochemical evidence that systemic cisplatin administration induces significant retinal toxicity in this Wistar rat model, corroborating and expanding upon the findings of previous studies. Retinal toxicity and vision loss constitute some of the most distressing and frequently encountered adverse effects of cisplatin in patients. The cytotoxic properties of cisplatin can induce a wide spectrum of ocular toxicities, including conjunctivitis, keratitis, optic neuritis, and reduced visual acuity. Cisplatin has also been associated with peripheral neuropathies that impair visual function, such as corneal neuropathy, which may progress to blindness in severe cases [85]. Consequently, numerous studies and authors have investigated cisplatin-induced retinal toxicity and have identified several significant electrophysiological and histopathological alterations [74,78,79,80].

The dosage and administration regimen of cisplatin vary substantially across studies in the literature, as summarized in Table 3. Nevertheless, regardless of the administered dose, the frequency of treatment, or the species of experimental animal used, several retinal lesions appear consistently. Macroscopically and under light microscopy, the retina typically presents with marked edema [70,73,74,75], whereas hematoxylin-eosin-stained sections reveal pronounced eosinophilia of retinal cells [76]. Across individual retinal layers, investigators have generally reported severe structural alterations [74], irregularity [73] and disorganization [70]. Furthermore, the GCL, as well as the INL and ONL, appear profoundly disorganized and edematous, with focal areas of degeneration within both the IPL and OPL [70,73]. Specifically, the GCL exhibits intense gliosis, likely reflecting a neuroglial response to cisplatin-induced cytotoxicity [71], along with reduced nuclear density, a substantial overall decrease in ganglion cell number and congestion of capillary vessels accompanied by infiltration of neutrophilic polymorphonuclear leukocytes [73]. Moreover, while Ibrahim and colleagues (2019) administered a comparatively lower intraperitoneal dose of cisplatin, the lowest among similar studies, they reported that the nuclei of the ganglion cell layer remained largely normal, with no evidence of gliosis described in other studies, thereby providing direct support for the dose-dependent cytotoxicity of cisplatin even at the level of the retina [75]. With respect to the photoreceptor layer, even Ibrahim et al. (2019), despite the low dosage administered, reported severe fragmentation of the photoreceptors [75]. Degeneration and disorganization of cones and rods have also been documented in other studies employing higher cisplatin doses [70,71]. With respect to the nerve fiber layer and the optic nerve, Raheem et al. (2023) [71] reported severe ischemic injury to the optic nerve, indicative of demyelination, along with pronounced infiltration by inflammatory cells, likely lymphocytes or microglia, which formed clustered aggregates. They also reported vascular congestion and a substantial reduction in the number of astrocytes and oligodendrocytes, the latter being responsible for the formation of the myelin sheath [71]. Finally, another important parameter for assessing cisplatin-induced toxicity is total retinal thickness. Most studies report a statistically significant increase in overall retinal thickness, primarily attributable to swelling of the outer and inner nuclear layers and the plexiform layers [73,74,76]. However, Polat et al. (2023) reported a marked reduction in total retinal thickness, likely reflecting degeneration and loss of neural elements [70].

The present experimental study employed a lower per-dose amount of cisplatin (3.5 mg/kg IP) than most published protocols but administered it over a five-day schedule that more closely resembles clinical chemotherapy regimens for a wide range of malignancies. As a result, each animal received a cumulative dose of 17.5 mg/kg, which is higher than that used in many single-dose studies. The histological profile observed in Group A, characterized by retinal edema, ganglion cell degeneration and extensive photoreceptor damage, aligns with the “mitochondrial hypothesis” of platinum toxicity. Cisplatin is known to accumulate in mitochondria, where it forms adducts with mitochondrial DNA (mtDNA). Unlike nuclear DNA, mtDNA lacks robust nucleotide excision repair mechanisms, making it exceptionally susceptible to damage. The resulting impairment of mitochondrial transcription leads to the synthesis of defective electron transport chain subunits, uncoupling of oxidative phosphorylation, and a surge in superoxide radical production [73,74,76,77]. The ultrastructural data (TEM) obtained in this study offer a granular view of this process, which has rarely been reported in previous light microscopy-based studies. The observation of swollen mitochondria with cristolysis in the photoreceptor inner segments provides direct visual confirmation of mitochondrial permeability transition pore (mPTP) opening. This event is a critical tipping point in cell death, leading to the loss of the mitochondrial membrane potential, cessation of ATP synthesis, and the release of proapoptotic factors such as cytochrome c into the cytosol. Given the immense metabolic requirement of photoreceptors to maintain the “dark current” (the constant influx of Na^+^ and Ca^2+^ in the absence of light), even a marginal compromise in mitochondrial efficiency can lead to rapid energy failure and cell death. Furthermore, the significant gliosis and eosinophilia observed in the GCL suggest that RGCs are also primary targets. RGCs are central nervous system neurons that, once lost, do not regenerate. The upregulation of TGF-β2 in this layer is consistent with a reactive glial response. Müller cells, the primary glia of the retina, become activated by neuronal stress, upregulate intermediate filaments (GFAP) and secrete TGF-β. While initially protective, chronic gliosis leads to the formation of a glial scar, which can physically and chemically impede potential regenerative processes.

### 4.2. The Neuroprotective Efficacy of Selenium

To mitigate the extensive retinal and systemic toxicity of cisplatin, several agents have been proposed as potential protective or preventive interventions, including melatonin [70], azilsartan [71], coenzyme Q10 [73], lutein [75,80], astaxanthin [76], pycnogenol [79], fish oils [77], hesperidin [74] and selenium [72]. The results from Groups B and D validate the hypothesis that selenium supplementation can attenuate cisplatin-induced retinal damage. The protective mechanism is hypothesized to involve restoration of antioxidant enzymatic capacity, though direct measurement of antioxidant markers was not performed in the present study. Selenium is the obligate cofactor for Glutathione Peroxidase 4 (GPx4), an enzyme uniquely capable of reducing phospholipid hydroperoxides within cell membranes [69]. In the context of cisplatin toxicity, where lipid peroxidation of the polyunsaturated fatty acid-rich photoreceptor disks is a primary driver of damage, GPx4 activity is critical. By maintaining high systemic levels of selenium, the treatment likely ensures maximal saturation of GPx enzymes, allowing the retina to quench the ROS surge induced by cisplatin.

A central finding of this study is the divergence in outcomes based on the route of selenium administration. Group B (Selenium IP) was associated with greater morphological and ultrastructural preservation of mitochondria and photoreceptor disks on qualitative TEM assessment, but maintained elevated IL-6 immunohistochemical scores. This paradox may be explained by the pharmacokinetics of IP administration. IP delivery allows for rapid absorption into the systemic circulation via the portal and systemic venous systems, bypassing the initial rate-limiting step of gastric emptying. This may result in higher peak plasma concentrations of selenium species reaching retinal tissue more rapidly during the acute phase of cisplatin injury, although this inference requires confirmation through pharmacokinetic measurements, as no pharmacokinetic measurements were performed in this study. The persistence of mild IL-6 expression in this group, rather than being a sign of failure, might actually contribute to the superior structural outcome. As noted in the introduction, IL-6 has neurotrophic properties via the gp130 signaling pathway, promoting RGC survival and axonal regeneration. One possible interpretation is that the intermediate IL-6 levels in Group B reflect a neurotrophic, pro-survival signaling response in cells whose viability is maintained by the structural protection afforded by IP selenium, whereas the massive IL-6 spike in Group A represents pathological, runaway inflammation. This hypothesis is consistent with the established dual role of IL-6 in neural tissue repair but requires functional validation.

Conversely, oral administration resulted in substantial suppression of IL-6 and TGF-β2, effectively normalizing the inflammatory profile to control levels. However, the degree of ultrastructural damage to the mitochondria was greater than that in the IP group. Oral selenium is subject to hepatic metabolism. Absorbed selenium is transported to the liver, where it is prioritized for the synthesis of selenoprotein P (Sepp1) before being released into the systemic circulation. This regulation ensures homeostasis but may dampen the bolus effect needed to counteract an acute toxic insult, such as a high-dose cisplatin injection. Furthermore, the near-complete suppression of IL-6 immunohistochemical expression in Group D might have been detrimental, depriving the stressed retina of the necessary neurotrophic signals required for repair, leading to the observed persistence of mitochondrial swelling.

The differential outcomes between IP and oral selenium may be mechanistically explained by the SELENOP-dependent selenium transport system. SELENOP, produced primarily by hepatocytes, is the principal selenium carrier in plasma and delivers selenium to peripheral tissues via receptor-mediated endocytosis through ApoER2 and megalin [54,62,63,64,65,66]. Under conditions of oral selenium intake, absorbed selenium is first incorporated into hepatic SELENOP before redistribution, a process that maintains selenium homeostasis but may limit the peak selenium concentrations achievable in extrahepatic tissues during acute insults. In contrast, intraperitoneal administration may partially bypass this hepatic sequestration, allowing selenium species to enter the systemic circulation more rapidly and potentially achieve higher transient retinal concentrations during the critical window of cisplatin-induced oxidative stress. Importantly, however, this SELENOP-based interpretation remains a plausible hypothesis in the context of the present study, as neither plasma nor tissue selenium levels nor SELENOP concentrations were measured. Direct demonstration of route-dependent selenium pharmacokinetics in retinal tissue would require targeted assessments of systemic and tissue selenium or SELENOP levels, which should be prioritized in future studies.

Our results support the findings of Akşit et al. (2016), who showed the protective effect of selenium on retinal carbohydrate residues, but we extend this by demonstrating the ultrastructural basis of this protection and the critical importance of the delivery route [72].

### 4.3. Clinical Implications and Future Directions

The translation of these findings to clinical practice requires careful consideration. The observation that IP selenium was associated with greater ultrastructural preservation in this morphological and immunohistochemical study suggests that, in the acute setting of chemotherapy, parenteral administration may be needed to achieve sufficient ocular tissue concentrations for structural protection. Similarly, the observation that oral selenium was associated with lower semi-quantitative IHC scores for IL-6 and TGF-β2 may suggest a role in modulating the cytokine-level inflammatory response, which might be excellent for long-term maintenance and suppression of chronic inflammation, but insufficient for acute cytoprotection against highly potent alkylating agents.

Future research should focus on correlating these structural findings with functional outcomes via ERG to confirm that preserved morphology translates to preserved vision, investigating whether higher oral doses can mimic the protective effects of parenteral administration without inducing selenium toxicity and exploring whether combining selenium with other antioxidants, such as zinc, magnesium and vitamin D, can offer synergistic protection.

### 4.4. Limitations

Despite the integrated immunohistochemical and ultrastructural evidence presented, several important limitations must be acknowledged. First, no pharmacokinetic data were collected: plasma and retinal tissue selenium concentrations, SELENOP levels, or glutathione peroxidase (GPx) activity were not measured. Additionally, no gp130 pathway activation, no downstream STAT3 phosphorylation, no Müller cell-specific activation markers, such as GFAP upregulation, and no retinal ganglion cell survival data were collected. Consequently, the interpretation that route-dependent differences in outcomes are attributable to differential bioavailability and inter-animal variability in gastrointestinal absorption remains a plausible hypothesis rather than a demonstrated mechanism. Future studies should incorporate plasma selenium, SELENOP, and tissue selenium measurements at multiple time points. Second, no functional retinal assessment, such as electroretinography, was performed. While morphological preservation strongly suggests maintained function, structural integrity does not guarantee functional preservation, and subclinical functional deficits may exist despite apparently intact ultrastructure. Third, the immunohistochemical assessment used a semi-quantitative ordinal scoring system (0–3). Although this approach is standard and was applied by two blinded independent observers, it is inherently subjective and provides limited resolution compared to automated digital image analysis or densitometry. The absence of formal inter-rater reliability statistics represents an additional limitation. Fourth, ultrastructural findings were assessed qualitatively from representative TEM images; a formal semi-quantitative TEM injury score, comprising grading mitochondrial swelling, cristolysis, or disk fragmentation, was not applied. Fifth, only male animals were used, precluding assessment of sex-dependent differences in selenium metabolism or cisplatin toxicity. Sixth, the assessment was confined to a single two-week post-treatment time point, which precludes evaluation of long-term retinal recovery, delayed neurotoxicity, or chronic selenium effects. Seventh, the potential for selenium to interfere with the antitumor efficacy of cisplatin was not assessed in this non-tumor-bearing model; this interaction must be evaluated before any clinical translation. Eighth, while the Wistar rat model provides anatomical similarity to the human ocular system, IP administration in rodents does not directly translate to IV administration in humans, as IP delivery involves peritoneal absorption dynamics distinct from direct venous infusion. Ninth, the control group (Group C) did not receive vehicle-matched administrations equivalent to those used for the intraperitoneal injection (Groups A, B) or oral gavage (Group D) procedures. While all the animals were handled at a comparable frequency and under identical environmental conditions, procedural stress from repeated gavage or injection cannot be entirely excluded as a confounder for cytokine expression in the treatment groups.

## 5. Conclusions

This experimental investigation demonstrated that systemic cisplatin administration induces severe subcellular retinal damage in Wistar rats, characterized by mitochondrial swelling with cristolysis and photoreceptor disk fragmentation, accompanied by the upregulation of IL-6 and TGF-β2 expression. Selenium supplementation, administered as sodium selenite (2.73 mg/kg/day; cumulative dose of 60 mg/kg over 22 days), attenuated cisplatin-induced retinal changes through both administration routes but with distinct outcome profiles. Intraperitoneal selenium was associated with greater preservation of photoreceptor and mitochondrial ultrastructure on qualitative TEM assessment, whereas oral selenium was associated with lower semi-quantitative IHC scores for IL-6 and TGF-β2, reaching near-control levels. This dissociation between cytokine suppression and structural preservation highlights the complexity of the retinal response to combined cisplatin–selenium exposure and suggests that the route of administration may influence the neuroprotective profile of selenium. These preliminary morphological and immunohistochemical findings warrant further investigations incorporating pharmacokinetic measurements (plasma or tissue selenium, SELENOP), functional retinal outcomes (ERG), and assessments of tumor-response interactions before clinical translation can be considered.

## Figures and Tables

**Table 1:** Descriptive Statistics for IL-6 Immunohistochemical Scores.

Group	N	Median	IQR	Mean	SD	Range	Skewness
Control	10	0.00	0.00–0.00	0.10	0.32	0–1	3.16
Group A (Cis)	10	2.00	2.00–2.25	2.20	0.42	2–3	1.78
Group B (IP Se)	10	1.50	1.00–2.00	1.50	0.53	1–2	0.00
Group D (Or Se)	10	0.00	0.00–1.00	0.40	0.52	0–1	0.48

**Table 2:** Descriptive Statistics for TGF-β2 Immunohistochemical Scores.

Group	N	Median	IQR	Mean	SD	Range	Skewness
Control	10	0.00	0.00–0.00	0.10	0.32	0–1	3.16
Group A (Cis)	10	1.00	1.00–2.00	1.30	0.48	1–2	1.04
Group B (IP Se)	10	0.00	0.00–1.00	0.40	0.52	0–1	0.48
Group D (Or Se)	10	0.00	0.00–1.00	0.30	0.48	0–1	1.04

**Table 3:** Summary of Experimental Studies Using Animal Models to Investigate Cisplatin-Induced Retinal Toxicity.

Author, Year	Cisplatin Regimen	Animal Model Used
Raheem et al., 2023 [71]	7 mg/kg IP (one dose)	Male Wistar rats 240–300 g
Polat et al., 2023 [70]	7 mg/kg IP (one dose)	Male Wistar rats 200–300 g
Sunar et al., 2021 [73]	2,5 mg/kg IP (for 14 days)	Male Wistar rats 290–300 g
Ibrahim et al., 2019 [75]	1 mg/kg IP (twice a week for 15 days)	Male New Zealand rabbits 1500–2000 g
Fındık et al., 2019 [76]	16 mg/kg IP (one dose)	Male Sprague Dawley rats 264.83 ± 7.39 g
Gul Baykalir et al., 2018 [77]	7 mg/kg IP (one dose)	Male Spraque Dawley rats 250–300 g
Akşit et al., 2016 [72]	16 mg/kg IP (for 3 days)	Male Wistar rats 250–300 g
Polat et al., 2016 [74]	7 mg/kg IP (one dose)	Male Spraque Dawley rats 250–300 g

## Data Availability

The original contributions presented in this study are included in the article. Further inquiries can be directed to the corresponding authors.

## Supplementary Materials

The following supporting information can be downloaded at: https://www.mdpi.com/article/10.3390/nu18081236/s1. Table S1: ARRIVE 2.0 checklist; Figure S1: CONSORT-style flow diagram of animal allocation and analysis.
