# Supplementary material for: Protective Efficacy of Selenium in Cisplatin-Induced Retinal Toxicity: An Experimental Immunohistochemical and Ultrastructural Analysis
Source: Nutrients. 2026 Apr 14;18(8):1236. doi: 10.3390/nu18081236 (PMC13119095; doi:10.3390/nu18081236)
Supplement: Supplementary file 1 [file nutrients-18-01236-s001.zip › nutrients-4102879-Figure S1.pdf]

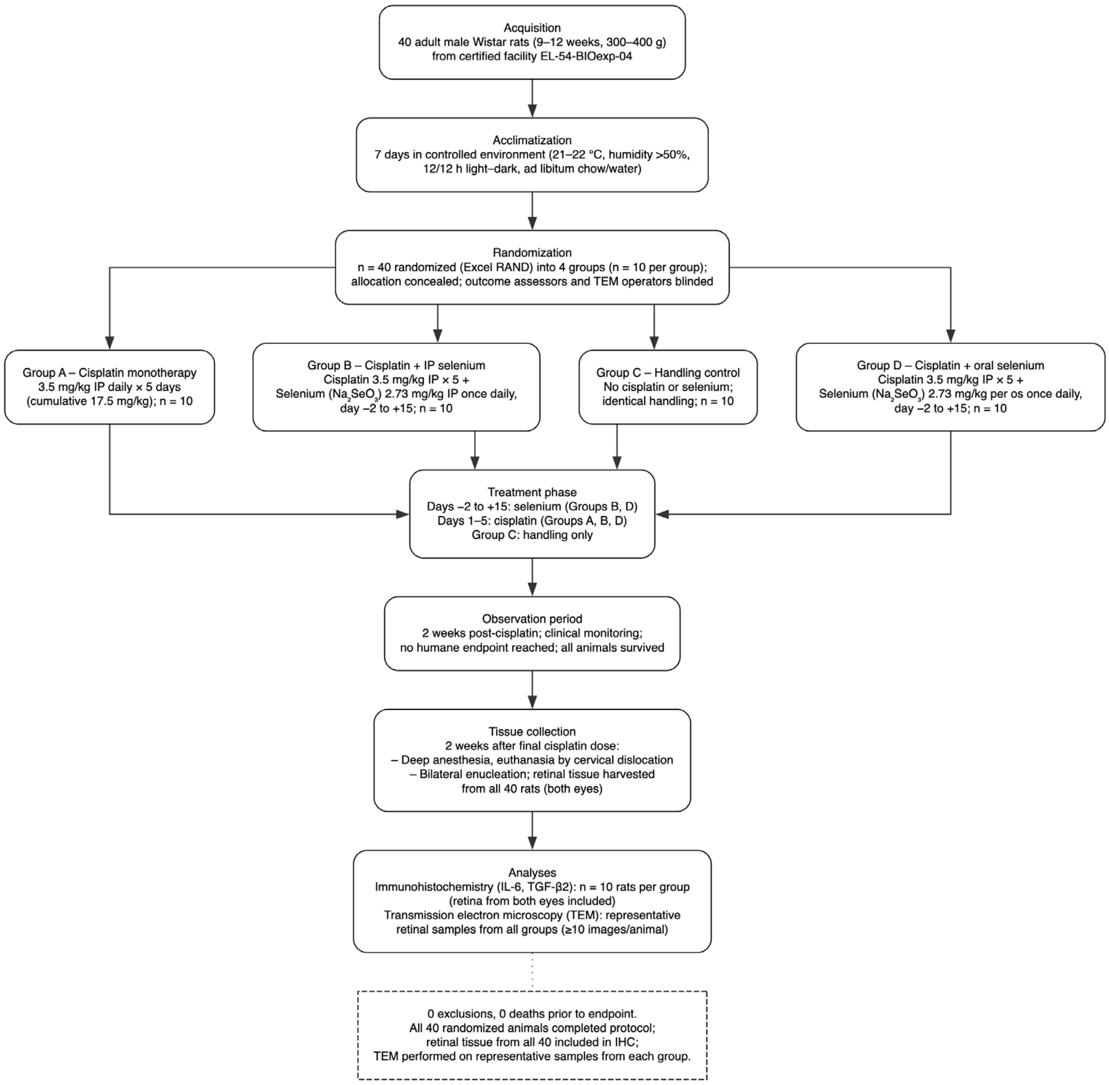

Supplementary Figure S1. CONSORT-style flow diagram of animal allocation and analysis. Forty adult male Wistar rats (9–12 weeks, 300–400 g) were obtained from a certified facility (EL-54-BIOexp-04), acclimatized for 7 days, and randomized (Excel RAND) to four groups (n = 10 per group): Group A, cisplatin monotherapy; Group B, cisplatin plus intraperitoneal selenium; Group C, handling control; Group D, cisplatin plus oral selenium. Selenium (cumulative 60 mg/kg as sodium selenite) was administered once daily from 2 days before until 15 days after the cisplatin regimen, while cisplatin (3.5 mg/kg IP) was given once daily for 5 days (cumulative 17.5 mg/kg). Retinal tissue was collected 2 weeks after the final cisplatin dose for immunohistochemistry (IL-6, TGF-β2; n = 10 per group) and transmission electron microscopy. No animals met humane endpoints; no animals or data points were excluded.
